# The Impact of Acute Matriptase Inhibition in Hepatic Inflammatory Models

**DOI:** 10.1155/2016/6306984

**Published:** 2016-08-24

**Authors:** Judit Pomothy, Gergely Szombath, Patrik Rokonál, Gábor Mátis, Zsuzsanna Neogrády, Torsten Steinmetzer, Erzsébet Pászti-Gere

**Affiliations:** ^1^Department of Pharmacology and Toxicology, University of Veterinary Medicine, István u. 2, Budapest 1078, Hungary; ^2^Department of Physiology and Biochemistry, University of Veterinary Medicine, István u. 2, Budapest 1078, Hungary; ^3^Faculty of Pharmacy, Institute of Pharmaceutical Chemistry, Philipps University Marburg, Marbacher Weg 6, 35037 Marburg, Germany

## Abstract

*Purpose*. Dysfunction of matriptase-2 can be involved in iron regulatory disorder via downregulation of hepcidin expression. In the present study, we investigated the effects of 3-amidinophenylalanine-derived matriptase inhibitors on porcine hepatic inflammatory cell models.* Methods*. Hepatocyte-Kupffer cell cocultures (ratio of 2 : 1 and 6 : 1) were treated with four structurally related matriptase inhibitors at 50 *μ*M. Cell cytotoxicity and relative expressions of IL-6 and IL-8 and the levels of hepcidin were determined by MTS and porcine-specific ELISA. The extracellular H_2_O_2_ contents were analyzed by Amplex Red method.* Results*. Matriptase inhibitors at 50 *µ*M for 24 h did not increase cell death rate. The elevated ROS production observed after short-term application of inhibitor MI-441 could be correlated with lowered hepcidin expression. MI-460 could significantly enhance hepcidin levels in the supernatants of cocultures (by 62.21 ± 26.8% in hepatocyte-Kupffer cell, 2 : 1, and by 42.6 ± 14.3% in hepatocyte-Kupffer cell, 6 : 1, cocultures, resp.). No significant changes were found in IL-6 and IL-8 levels in cocultures exposed to matriptase inhibitors.* Conclusions*. Based on* in vitro* findings, administration of MI-460 via modulation of hepcidin expression without cytotoxic and oxidative stress inducing properties might be a reliable alternative to treat iron overload in human and veterinary clinical practice.

## 1. Introduction

Several type II transmembrane serine proteases (TTSPs) are associated with cell surface proteolysis and can be divided into four subfamilies such as human airway trypsin-like protease (HAT)/differentially expressed in squamous cell carcinoma (DESC), hepsin/transmembrane protease, serine 2 (TMPRSS), corin, and matriptase (MT). They also play crucial roles in tightly regulated physiological processes [1]. Expression of matriptase-1 (MT-1) is not limited to any particular organ [2], whereas matriptase-2 (MT-2) mainly accumulates in the liver in both human and mouse, which reveals its tissue-specific functions [3].

Consequently, deregulated MT-1 and MT-2 activities have been linked to various pathological conditions. It was reported that MT-1 overproduction in osteoarthritis could promote the induction of cartilage destruction [4]. MT-1 activity is also involved in tumorigenesis of various cancer types and promotes the progression of malignant transformations via cleavage and activation of substrates like hepatocyte growth factor/scatter factor [5–7].

MT-2, also known as TMPRSS6, is highly expressed in the liver and it negatively affects the production of the hepatic hormone hepcidin, the main regulator of systemic iron homeostasis through the interaction with the bone morphogenetic protein coreceptor hemojuvelin (HJV) [8–10]. After binding, MT-2 degrades and reduces cell surface HJV and prevents hepcidin from being expressed via induction through bone morphogenetic protein 6 (BMP6) [9]. Active HJV facilitates transcription of hepcidin responsible for iron transport to the bloodstream. This mechanism is impaired in iron-refractory iron deficiency anaemia due to the hepcidin-dependent cellular iron accumulation and loss of MT-2 proteolytic function as a result of genetic mutation [11, 12].

The elevated production rate of hepcidin triggered by high iron level can lower the iron absorption from the duodenum and reduce the iron release from the iron storages thus stabilizing the finely adjusted physiological iron concentration [13, 14]. The hepcidin secretion in the liver can be elevated due to the infections and inflammatory disorders, which can be facilitated by the proximity of pathogens-sensing Kupffer cells, to which the body reacts with iron deficiency and anaemic conditions consequently. This host defensive mechanism in response to infectious diseases can provide significant reduction in the amount of iron essential for the survival of the microbes. In case of sustained iron deficiency, the hepatocytes produce less hepcidin; thus iron concentration increases again in the blood circulation. Other influencing factors such as bleeding, application of erythropoietin (EPO), and overproduction of reactive oxygen species (ROS) can also modulate the hepcidin expression significantly [15].

MT-1 and MT-2 are belonging to the TTSPs having an extracellular trypsin-like serine protease domain at the C-terminus, which can be blocked by 3-amidinophenylalanine-derived inhibitors. The most potent inhibitors contain N-terminal dichloro- or dimethoxy-biphenyl-3-sulphonyl group and inhibit MT-1 in the low one-digit nanomolar range, whereas MT-2 is less potently inhibited [16]. As mentioned above, MT-2 plays an important regulatory role in iron homeostasis; thus this enzyme may act as a pharmacological target in the treatment of the systemic iron overload (hemochromatosis). This approach seems to be supported by the fact that lack of functional MT-2 can result in high hepatic hepcidin expression, which results in iron-deficiency anaemia [8].

Appropriately established cell models can be used to mimic* in vivo* pathophysiological conditions such as inflamed liver thereby enabling the replacement of some animal experiments. A porcine primary hepatocyte-Kupffer cell coculture with variable ratios of cell types has been previously developed and characterized [17]. In this model, hepatocytes were cocultured with Kupffer cells in the ratio of 6 : 1 (HepK6) and 2 : 1 (HepK2) in an attempt to mimic hepatic inflammation with different severity. This system seems to be a proper tool to investigate certain molecular mechanisms within the inflamed liver, such as to assess the possible effects of MT-2 inhibition on the regulation of iron homeostasis in hepatic inflammation.

The main aim of this research was to explore the possible relationship between coculture model of hepatocytes and Kupffer cells of swine liver and selected drug candidates. In our study, the regulatory properties of four potent 3-amidinophenylalanine-derived MT-1 inhibitors (MI-432, MI-441, MI-460, and MI-461), which also possess a moderate inhibitory potency against MT-2, were elucidated* in vitro* in hepatic inflammatory models HepK2 and HepK6 cocultures to determine how MT-1/MT-2 modulation affects cell viability and physiological redox status, possibly being in connection with hepcidin production. The analysis of changes in the hepcidin expression was a prerequisite for exploring a link between the effect of MT-1/MT-2 suppression and iron homeostatic processes. As hepcidin-mediated disturbances in iron metabolism are often coupled to an inflammatory response, therefore, the expressions of the proinflammatory cytokines, IL-6 and IL-8, were also monitored.

## 2. Materials and Methods

### 2.1. Isolation and Cultivation of Hepatocytes and Kupffer Cells

Hepatocyte-Kupffer cell cocultures were freshly prepared from 15 kg weighing male pigs of the Hungarian Large White breed (obtained from Dunahyb Ltd., Fadd, Hungary) [17]. Briefly, hepatocytes and Kupffer cells were isolated by multistep perfusion and collagenase-mediated enzymatic digestion of the caudate lobe of the liver. Hepatocytes were sedimented and purified by a revised low-speed centrifugation (50 ×g, 75 s); Kupffer cells were obtained from the supernatants by Percoll gradient centrifugation (500 ×g, 20 min). The viability of both cell types was assessed by trypan blue exclusion test and revealed more than 90% viable cells in all cases.

**Figure 1:**
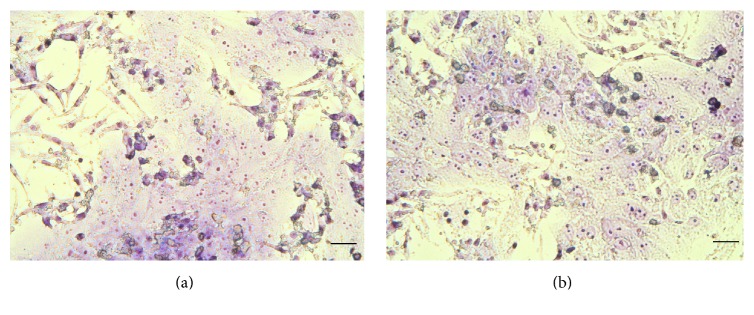
Giemsa staining of cocultures. HepK cell cocultures with the cell ratio of 2 : 1 (a) and 6 : 1 (b) (hepatocyte to Kupffer cell) after 24 h culturing following Giemsa staining (200x magnification). Scale bar = 50 *μ*m.

As nonparenchymal cells can attach faster, Kupffer cells were seeded first on previously collagen-coated (Type I, 10 *μ*g/cm^2^) 12-well Costar TC6 cell culture dishes (Corning International, Corning, NY, USA) and on 96-well microplates, the latter being used for MTS assays on cell viability. Hepatocytes and Kupffer cells were seeded on the same dishes in the appropriate cell ratio 6 : 1 (hepatocyte to Kupffer cell) for modelling moderate and 2 : 1 for more severe inflammation. The prepared cocultures were incubated under humid atmosphere at 37°C using 10% CO_2_, and culture medium was exchanged 6 h after seeding. Culturing of cells was performed in Williams medium E, supplemented with 10000 IU/mL penicillin, 10 mg/mL streptomycin, 2 mM glutamine, 10% FBS, and 0.2 IU/mL insulin. Fetal bovine serum (FBS) was applied during the first 24 h of culturing. Confluent monolayer cocultures were obtained after 24 h incubation (Figures 1(a) and 1(b)), being suitable for the further examinations.

### 2.2. Chemical Structures of Applied Matriptase Inhibitors

The structures of the used 3-amidinophenylalanine-derived inhibitors and their previously published *K*_*i*_ values against MT-1 and MT-2 [16] are summarized in Table 1. The compounds MI-432 and MI-441 are dibasic inhibitors containing a C-terminal 2-aminoethylpiperidide group, whereas the analogues MI-460 and 461 are more hydrophobic monobasic derivatives.

### 2.3. Exposure of HepK2 and HepK6 Cells to Matriptase Inhibitors

Stock solutions of the inhibitors were prepared with a concentration of 10 mM and kept at −20°C. Before treatment, confluent HepK2 and HepK6 cocultures were washed twice with plain medium. The solutions of the inhibitors in phenol red free Williams medium E at 50 *μ*M were prepared freshly prior to each experiment from the stock solutions. After incubation of the inhibitor for 2 h (in case of H_2_O_2_, IL-6 and IL-8, and hepcidin assays) or 24 h (in case of cell viability tests), the cocultures were washed twice with plain medium before being subjected to the subsequent procedures.

### 2.4. MTS Assay for Cell Viability

Influence of matriptase inhibitors in phenol red free Williams medium E on the viability of HepK2 and HepK6 cocultures was tested. Monolayer cocultures, grown on a 96-well plate for 24 h, were incubated with matriptase inhibitors for 24 h in treated groups. The control cells were incubated only with phenol red free Williams medium E. After removal of the medium and washing the cells 3-fold with phosphate-buffered saline (PBS), 20 *μ*L of CellTiter 96 aqueous one solution proliferation assay (MTS, Promega, Bioscience, Budapest, Hungary) containing tetrazolium compound and an electron coupling reagent, phenazine ethosulfate, was pipetted into each well of the 96-well assay plate containing the samples in 100 *μ*L of culture medium. The plate was incubated with dye for 2 h. Viability of HepK2 and HepK6 cocultures was measured at 490 nm using EZ Read Biochrom 400 microplate reader.

### 2.5. Extracellular H_2_O_2_ Measurement by the Amplex Red Method

Fluorescence ROS measurement of cell supernatant was based on the detection of H_2_O_2_ using the Amplex Red Hydrogen Peroxide Assay Kit (Invitrogen, Molecular Probes). In the presence of horseradish peroxidase (HRP), Amplex Red reacts with H_2_O_2_ in a 1 : 1 stoichiometry producing a highly fluorescent resorufin [18]. Following the exposure of cocultures to matriptase inhibitors (50 *μ*M, 2 h), the H_2_O_2_ concentrations in the medium were determined 6 h after completion of the treatment using a working solution of 100 *μ*M Amplex Red and 0.2 U/mL HRP. After 30 min incubation with the dye at room temperature, the quantitative H_2_O_2_ contents were measured using a Victor X2 2030 fluorometer (*λ*_ex_ = 560 nm; *λ*_em_ = 590 nm).

### 2.6. Cytokine Measurements by Enzyme-Linked Immunosorbent Assay

After 2 h inhibitor treatment (50 *μ*M), HepK2 and HepK6 cocultures were incubated in the medium for 6 h. Culture media were collected, centrifuged (245 ×g for 10 min at room temperature), and diluted to measure the IL-6 and IL-8 levels.

Changes in IL-6 and IL-8 concentrations relative to those in controls were determined by porcine-specific IL-6 (Sigma-Aldrich, St. Louis, MO, USA) and IL-8 (Abcam, Cambridge, UK) ELISA kits according to the manufacturer's instructions. The absorbance values were detected at 450 nm using EZ Read Biochrom 400 microplate reader (Biochrom Ltd., UK).

### 2.7. Hepcidin ELISA Measurements

Porcine ELISA kit for hepcidin was obtained from Cloud-Clone Corp. (Houston, Texas, USA), employing the competitive inhibition enzyme immunoassay technique. After 2 h inhibitor treatment (50 *μ*M), the HepK2 and HepK6 cell cocultures were incubated for 6 h and then the culture media were collected, centrifuged (1000 ×g for 20 min at room temperature), and diluted in PBS. The assay procedure was performed according to the manufacturer's guideline. After addition of the 3,3′,5,5′-tetramethylbenzidine (TMB) substrate solution for 15 min, the intensity of color developed is reverse proportional to the concentration of hepcidin in the sample. The absorbance values were detected at 450 nm using a EZ Read Biochrom 400 microplate reader (Biochrom Ltd., UK).

### 2.8. Statistical Analysis

For statistical evaluation R 2.11.1 software package (2010) was applied. Statistical significance of differences was assessed with one-sample Student's *t*-tests for assessment of relative values. Differences between absolute means were evaluated by one-way analysis of variance (one-way ANOVA) with* post hoc* Tukey test, where data were of normal distribution and homogeneity of variances was confirmed. Differences were considered significant if the *p* value was <0.05 marked with ^*∗*^ (^*∗∗*^ *p* < 0.01).

## 3. Results

### 3.1. Assessment of Cell Viability after Matriptase Inhibition

**Figure 2:**
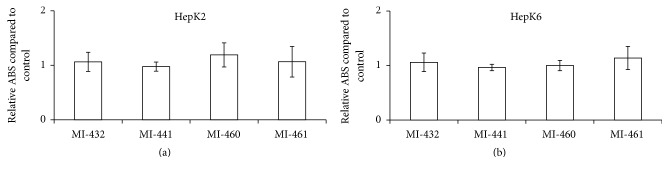
Lack of cytotoxic effects of matriptase inhibitors. (a) 24 h incubation of HepK2 cells with MI-432, MI-441, MI-460, or MI-461 at 50 *μ*M. Values represent average relative absorbance values of produced MTS formazan in metabolically active cells expressed in control values ± SEMs. No significant differences were found between control and cocultures exposed to matriptase inhibitors (*n* = 4). (b) HepK6 cocultures were treated with MI-432, MI-441, MI-460, or MI-461 at 50 *μ*M for 24 h. Relative absorbance values expressed in control values ± SEMs are indicated. No significant differences were found between control and treated cocultures (*n* = 4).

Cell cytotoxicity assays were performed to evaluate if the used inhibitors at 50 *μ*M concentration can cause significant cell death in HepK2 and HepK6 cocultures. It was found that the selected inhibitors can be used at 50 *μ*M concentration safely for 24 h in HepK2 (Figure 2(a)) and HepK6 cocultures (Figure 2(b)), and thus this concentration could be applied in further experiments to characterize the effects of MT-1/MT-2 modulation in* in vitro* inflammatory hepatic models.

### 3.2. Matriptase Inhibition-Induced Oxidative Stress

**Figure 3:**
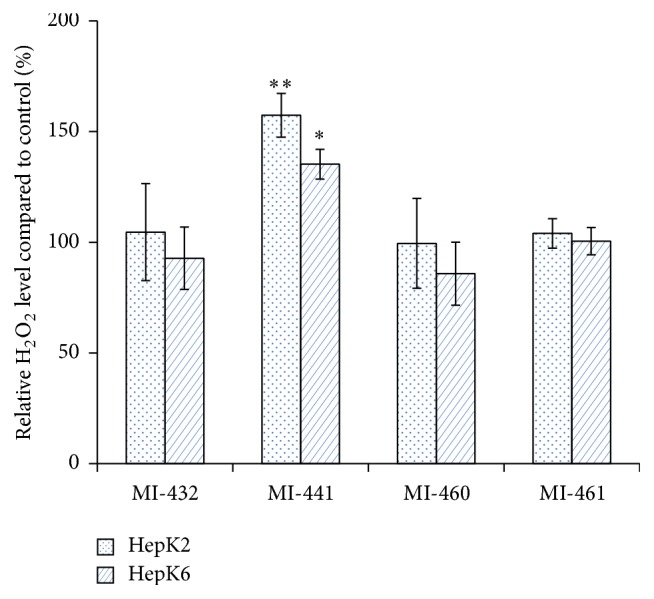
Extracellular ROS production of HepK2 and HepK6 cell cocultures control (Co) or exposed to matriptase inhibitors (MI-432, MI-441, MI-460, or MI-461) using Amplex Red method. Results are expressed as mean values relative to controls ± SEMs. Significant differences in fluorescence intensities were found between control and MI-441-treated coculture groups (^*∗*^ *p* < 0.05, *n* = 4, in HepK2 and ^*∗∗*^ *p* < 0.01, *n* = 4, in HepK6 samples).

Fluorescence intensities of both the control and treated samples were measured 6 h after a 2 h inhibitor application of matriptase inhibitors at 50 *μ*M. It was found that there was an increase in the extracellular H_2_O_2_ levels (Figure 3) causing a significant elevation in fluorescence, when inhibitor MI-441 was applied in HepK2 and HepK6 cocultures (*p* = 0.0147 and *p* = 0.00157, resp.). However, no significant perturbations in redox balance in case of HepK2 and HepK6 cocultures were observed after treatment with the other three inhibitors under identical conditions.

### 3.3. Determination of IL-6 and IL-8 Levels in the Supernatants of Hepk2 and Hepk6 Cocultures

**Figure 4:**
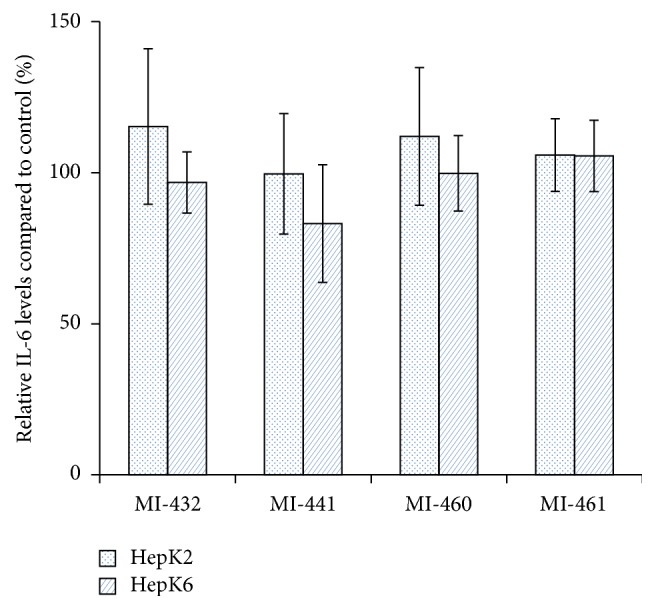
The effect of matriptase inhibition on IL-6 levels. The relative IL-6 levels after the HepK2 and HepK6 cell cocultures were treated with 50 *μ*M MI-432, MI-441, MI-460, and MI-461 for 2 h. The columns represent average relative absorbance values ± SEMs (*n* = 4). No significant differences were found (*p* > 0.05) between control and matriptase inhibitor-treated groups.

**Figure 5:**
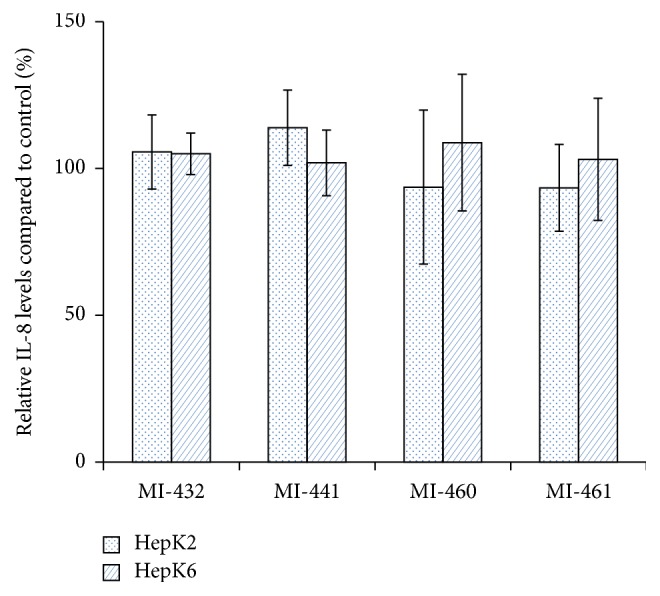
The effect of matriptase inhibition on IL-8 levels. The HepK2 and HepK6 cell cocultures were treated with 50 *μ*M MI-432, MI-441, MI-460, and MI-461 for 2 h. The columns represent average relative absorbance values ± SEMs (*n* = 4). No significant differences were found (*p* > 0.05) between control and matriptase inhibitor-treated groups.

After treatment of HepK2 and HepK6 cell cocultures with matriptase inhibitors at 50 *μ*M concentration for 2 h, the potential changes in IL-6 expressions were monitored in the cell supernatants. It was ascertained that none of the applied inhibitors could induce elevated production of IL-6 in HepK2 and in HepK6 cells (Figure 4). The relative absorbance values of IL-8 levels were also measured to determine the putative changes introduced by short-term administration of matriptase inhibitors. It was found that the applied compounds did not cause any significant alterations in the levels of IL-8 in the supernatants of HepK2 and HepK6 cocultures compared to those in control groups (Figure 5).

### 3.4. Analysis of Hepcidin Levels in the Supernatants of Hepk2 and Hepk6 Cell Cocultures

**Figure 6:**
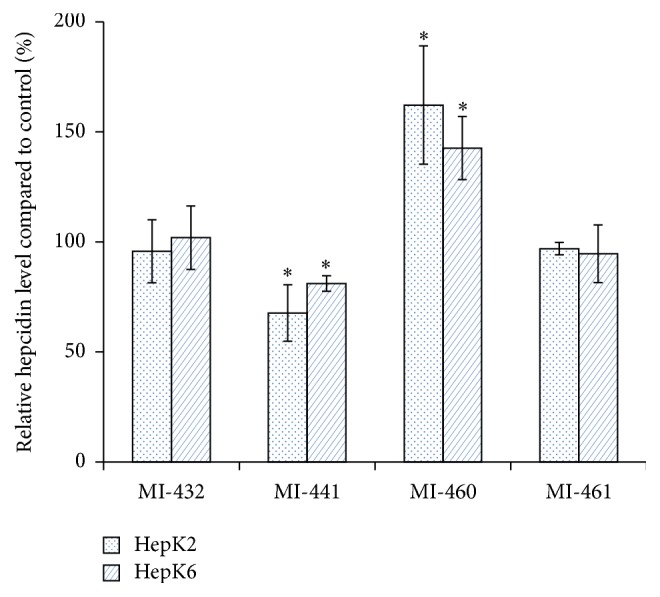
Changes in relative hepcidin levels after matriptase inhibition. Measurements were performed at 6 h after completion of 2 h 50 *μ*M MI-432, MI-441, MI-460, or MI-461 treatment. Each column represents the mean relative absorbance values ± SEMs (*n* = 3). The asterisk (^*∗*^ *p* < 0.05) shows statistical significances for results expressed in control percentage.

In HepK2 coculture it was found that MI-432 and MI-461 at 50 *μ*M for 2 h did not alter hepcidin levels significantly compared to those of control samples (*p* = 0.6571 and *p* = 0.2006, resp.). However, matriptase inhibition via application of MI-441 and MI-460 with the same incubation time and concentration has changed hepcidin levels in different ways. Treatment with MI-441 has lowered the hepcidin expression by 32.3 ± 12.8% (*p* = 0.0487), whereas it seems that compound MI-460 leads to an upregulation of hepcidin expression by 62.21 ± 26.8% (*p* = 0.0368) in the cell supernatants. Similar findings were obtained in HepK6 cell cocultures where MI-441 administration reduced the hepcidin levels (*p* = 0.0387) by 18.9 ± 3.5% and MI-460 elevated (*p* = 0.0114) the expression of hepcidin by 42.6 ± 14.3%. Application of MI-432 and MI-461 did not cause significant alterations in the levels of hepcidin in the supernatants of HepK6 compared to control samples (*p* = 0.8404 and *p* = 0.549, resp.) (Figure 6).

## 4. Discussion

Physiological plasma and cellular iron concentrations are provided via strict regulation of critical steps responsible for iron absorption, transport, storage, and recycling [19]. MT-2 plays a major role in inactivation of HJV thus reducing the expression of the liver-derived peptide hormone, hepcidin. Misregulation of hepcidin as a negative regulator of iron absorption has been found in many diseases such as the anemia of chronic disease, iron refractory iron deficiency anemia, cancer, hereditary hemochromatosis, and ineffective erythropoiesis such as *β*-thalassemia [20]. Other factors such as oxidative stress can also affect hepcidin levels. Experimental findings support that hepatitis C virus- (HCV-) induced oxidative stress inhibits hepcidin production offering a possible explanation for phenomenon of iron loading when this viral infection occurs [21, 22]. In the prevention and in the treatment of iron overload hepcidin agonists can represent therapeutical alternatives against several forms of the hemochromatosis. Small peptide analogues containing 7–9 amino acids, minihepcidins, were successfully applied in the regulation of plasma iron concentration in mice models [23]. It was reported that MT-2 could also affect the level of produced hepcidin. The loss of the serine protease domain of MT-2 in different species caused anaemia with increased hepcidin concentration and with low plasma iron level due to the reduced iron absorption [9, 10, 19]. MT-2 mutations in humans or lack of MT-2 in mice resulted in increased hepcidin expression and iron refractory iron deficiency anemia [8].

Elevated MT-1 levels could be found in epithelial cancers suggesting that the dysfunctional matriptase activity may contribute to tumor development and metastatic events. MT-1 could trigger the degradation of extracellular matrix components and intercellular structures thus enabling cell migration [5]. Moreover, also an aberrant expression of MT-2 is observed in several forms of cancers such as breast and prostate cancer [3, 24, 25]. However, more studies should be conducted to determine the efficacy of MT-1/MT-2 inhibitors on tumor formation and migration ability of cancerous cells more precisely based on the approach that matriptase inhibition might possess beneficial effect against tumor invasion and metastasis.

Suppression of MT-2 via application of 3-amidinophenylalanine compounds can be an effective pharmacological option in the effective treatment of hemochromatosis in the future [8, 12]. The safety of our used inhibitors at 50 *μ*M was confirmed since their 24 h long administration did not cause any significant changes in cell viability in HepK2 and HepK6 cocultures. Our data revealed that potent suppression of MT-2 activity via 2 h acute treatment of HepK2 and HepK6 cocultures with 50 *μ*M MI-460 could enhance hepcidin expression to great extent. This suggests that application of MI-460 can control dysregulated iron metabolism via increasing hepcidin level present mainly in several forms of systematic iron overload. In contrast, treatment with inhibitor MI-441 significantly decreased hepcidin levels, which might be explained by the fact that hepcidin synthesis can be affected via oxidative stress and as it was shown by us that extracellular H_2_O_2_ production was elevated by the addition of MI-441 in both cocultures. The other matriptase inhibitors (MI-432, MI-460, and MI-461), however, did not cause any significant changes in extracellular ROS production in these cocultures. In contrast, in our previous studies it was found that significant ROS release could be detected mainly after acute administration of MI-432 at 50 *μ*M in nontumorigenic intestinal porcine epithelial IPEC-J2 cell line via suppression of MT-1 enzyme activity. These apparent discrepancies between extracellular peroxide productions can be explained by various responsiveness of different cells such as IPEC-J2 cells and HepK cocultures towards matriptase inhibition [26]. As MI-432 and MI-461 did not show any acute effect on hepcidin regulation, thus further studies should be performed to clarify whether long-term applications of these compounds possess any impact on pathophysiological iron conditions.

Hepatic coculture systems consist of primary hepatocytes and Kupffer cells in a ratio of 6 : 1 and 2 : 1 and they can represent milder or severe inflammatory states of liver [17, 27]. Kupffer cells can be activated by bacterial endotoxins, which results in the elevated production of several proinflammatory cytokines such as IL-6 and IL-8. It was established that this coculture system can be used to examine chemical-induced inflammatory reactions such as shifting in cytokine profile (IL-6 and TNF-*α*) as a consequence of acute hepatocellular toxicity introduced by lipopolysaccharide and trovafloxacin administration [27]. In our study, 2 h inhibitor administration did not show any effects on IL-6 and IL-8 levels in HepK2 and HepK6 cocultures. This suggests that the hepatic inflammatory response is not influenced by the applied MT-1/MT-2 inhibitors.

In conclusion, taken together, the fact that MT-1 and MT-2 are membrane anchored serine proteases on the cell surface with relatively convenient accessibility for drugs and the downregulation of excessive matriptase activities can compensate severe pathological perturbations, further development and advanced biomolecular screening of more selective MT-1 and MT-2 inhibitors can be of high pharmacological and therapeutical importance.

## Figures and Tables

**Table 1:** Chemical structures and *K*_*i*_ values of the used 3-amidinophenylalanine-derived inhibitors towards matriptase-1 (MT-1), matriptase-2 (MT-2), and thrombin.

Number	Structure	*K*_*i*_ (*µ*M)		
		MT-1	MT-2	Thrombin
MI-432	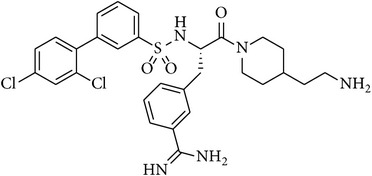	0.002	0.11	0.02

MI-441	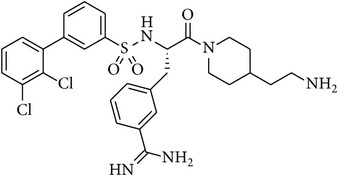	0.011	0.21	0.02

MI-460	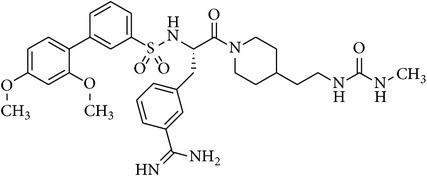	0.0018	0.18	0.0008

MI-461	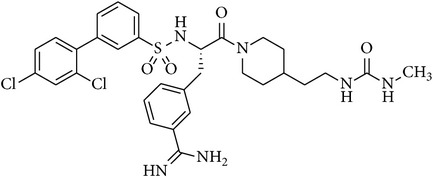	0.0009	0.073	0.0002
